# Supplementary material for: Open-Source Platform for Kinematic Analysis of Mouse Forelimb Movement
Source: bioRxiv. 2024 Apr 15:2024.01.31.578239. Preprint. [Version 2] doi: 10.1101/2024.01.31.578239 (PMC11042209; doi:10.1101/2024.01.31.578239)
Supplement: Supplementary file 1 [file media-1.pdf]

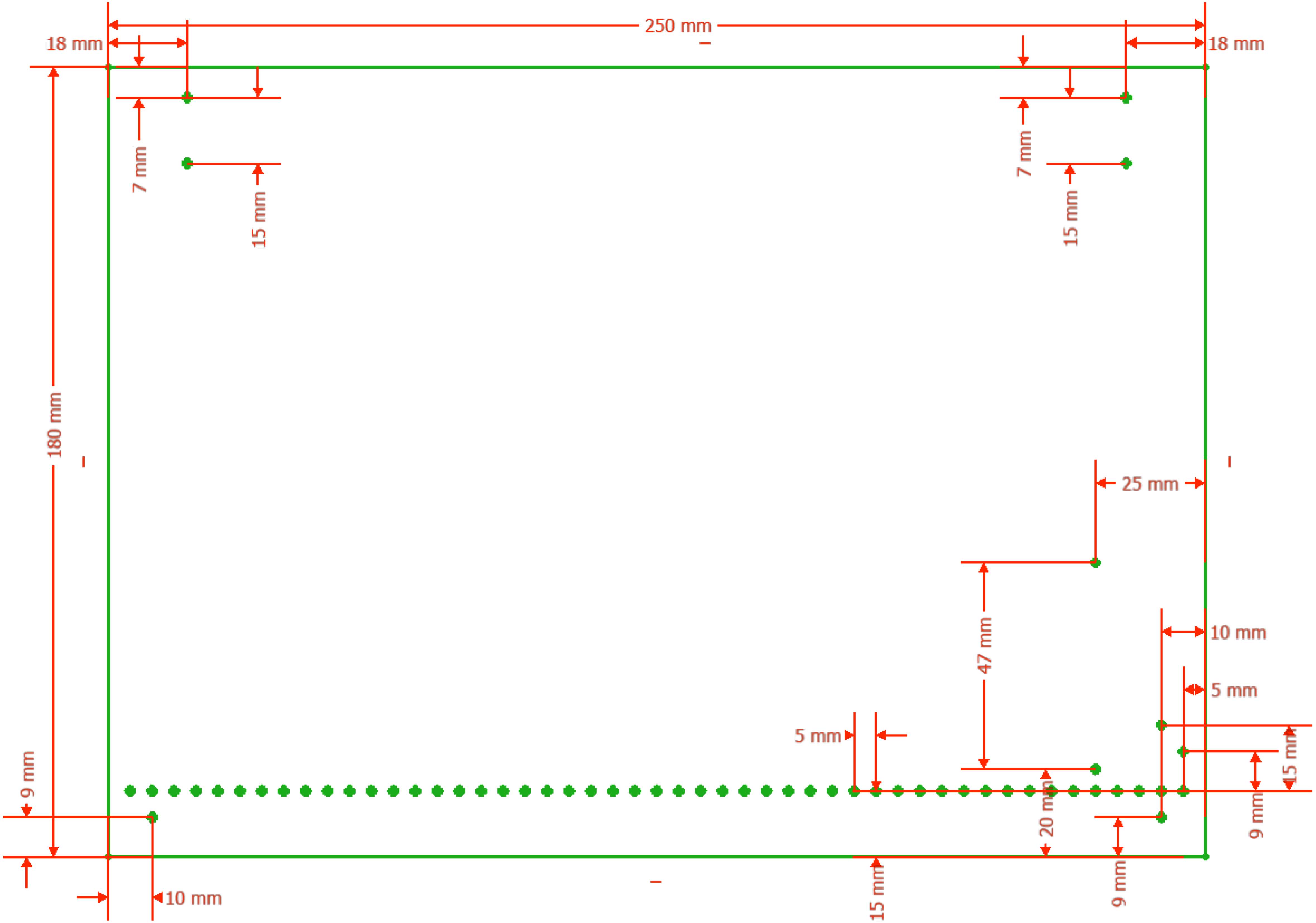

250 mm
18 mm
18 mm
7 mm
15 mm
7 mm
15 mm
180 mm
25 mm
47 mm
10 mm
5 mm
5 mm
15 mm
9 mm
20 mm
9 mm
15 mm
9 mm
10 mm
