# Supplementary material for: Open-Source Platform for Kinematic Analysis of Mouse Forelimb Movement
Source: bioRxiv. 2024 Apr 15:2024.01.31.578239. Preprint. [Version 2] doi: 10.1101/2024.01.31.578239 (PMC11042209; doi:10.1101/2024.01.31.578239)
Supplement: Supplementary file 3 [file media-3.pdf]

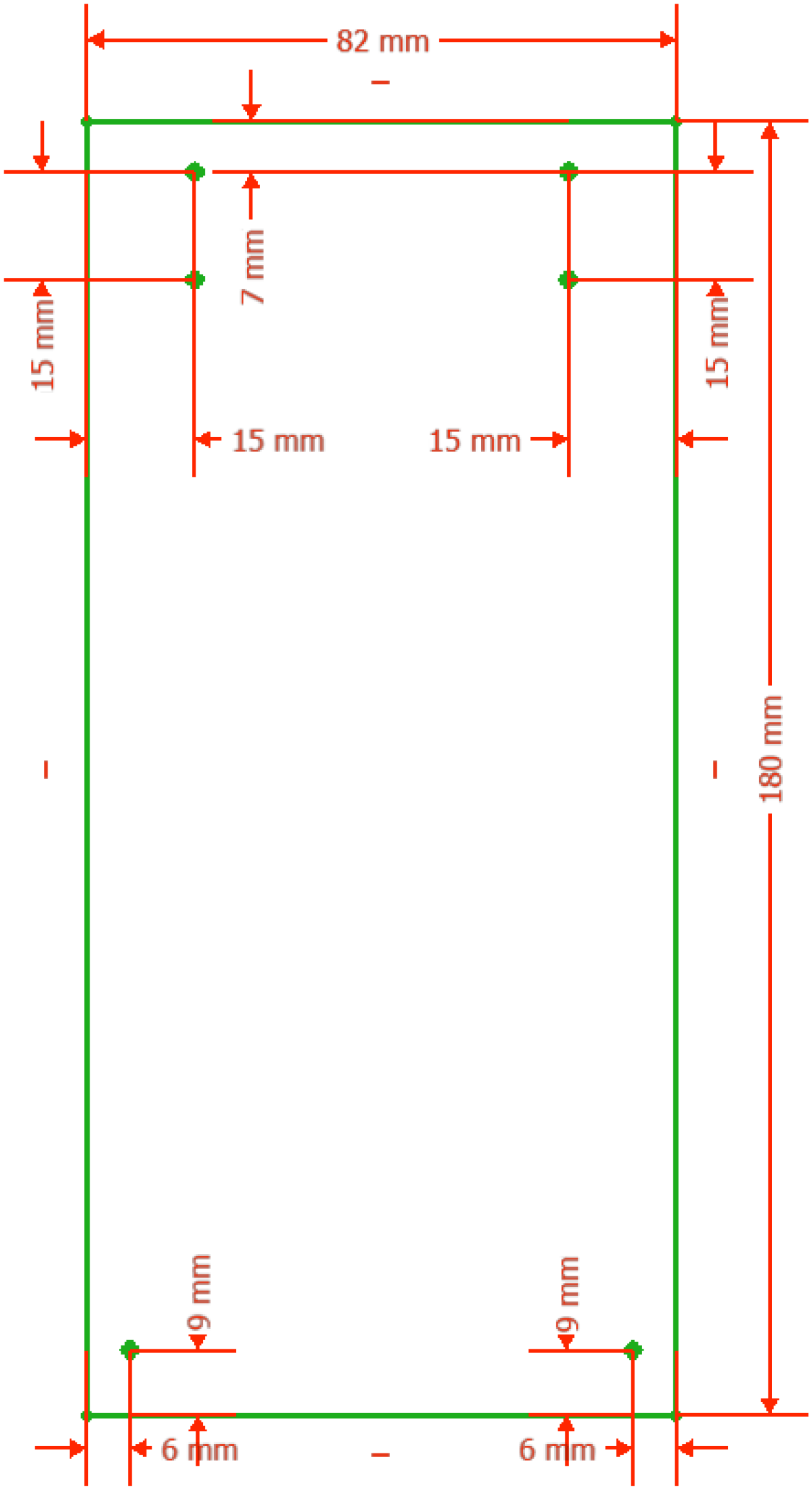
82 mm
7 mm
15 mm
15 mm
15 mm
15 mm
180 mm
9 mm
9 mm
6 mm
6 mm
