# Supplementary material for: Open-Source Platform for Kinematic Analysis of Mouse Forelimb Movement
Source: bioRxiv. 2024 Apr 15:2024.01.31.578239. Preprint. [Version 2] doi: 10.1101/2024.01.31.578239 (PMC11042209; doi:10.1101/2024.01.31.578239)

Main components:

1 – control buttons
2 – 4pin connector to 4
3 – 10pin connector to 6
4 – 4pin socket
5 – stepper motor socket to 8
6 – 10pin Arduino socket
7 – 10pin output socket
8 – feeder stepper motor
9 – beam sensor
10 – touch sensors

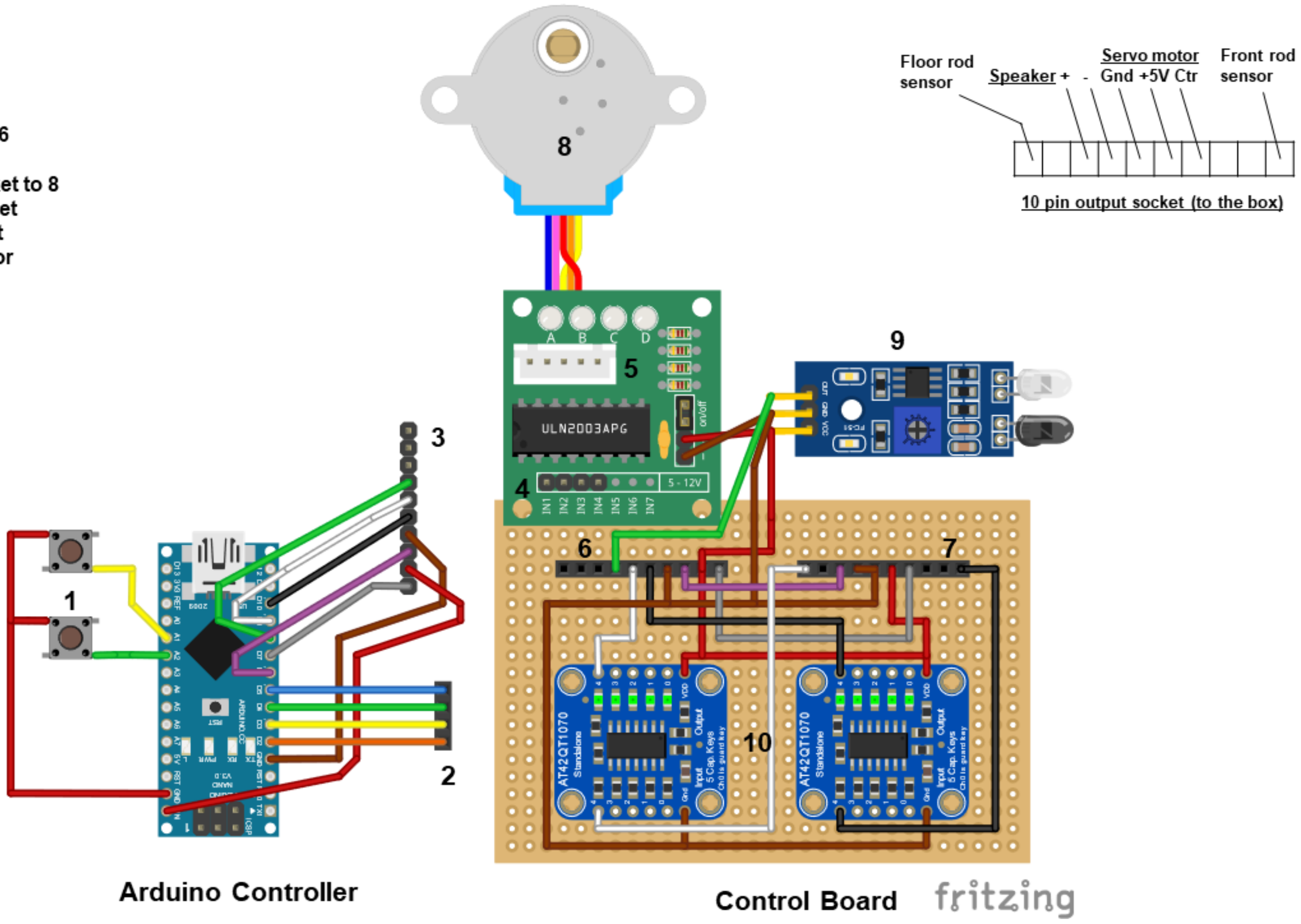

Supplement: Supplementary file 4 [file media-4.pdf]
